# Antifungal Agents Based on Chitosan Oligomers, ε-polylysine and *Streptomyces* spp. Secondary Metabolites against Three Botryosphaeriaceae Species

**DOI:** 10.3390/antibiotics8030099

**Published:** 2019-07-20

**Authors:** Laura Buzón-Durán, Jesús Martín-Gil, Eduardo Pérez-Lebeña, David Ruano-Rosa, José L. Revuelta, José Casanova-Gascón, M. Carmen Ramos-Sánchez, Pablo Martín-Ramos

**Affiliations:** 1 Departamento de Ingeniería Agroforestal, ETSIIAA, Universidad de Valladolid, Avenida de Madrid 44, 34004 Palencia, Spain; 2 Instituto Tecnológico Agrario de Castilla y León, Unidad de Cultivos Leñosos y Hortícolas, Ctra. De Burgos km 119, Finca Zamadueñas, 47071 Valladolid, Spain; 3 Departamento de Microbiología y Genética, Facultad de Biología, Universidad de Salamanca, Campus Miguel de Unamuno, C/ Donantes de Sangre, s/n, 37007 Salamanca, Spain; 4 Instituto Universitario de Investigación en Ciencias Ambientales de Aragón (IUCA), EPS, Universidad de Zaragoza, Carretera de Cuarte, s/n, 22071 Huesca, Spain; 5 Servicio de Microbiología y Parasitología, Hospital Universitario Rio Hortega, SACYL, Calle Dulzaina, 2, 47012 Valladolid, Spain

**Keywords:** *Botryosphaeria dothidea*, conjugate complexes, *Diplodia seriata*, grapevine trunk diseases, *Neofusicoccum parvum*

## Abstract

Grapevine trunk diseases (GTDs) are a major threat to the wine and grape industry. The aim of the study was to investigate the antifungal activity against *Neofusicoccum parvum*, *Diplodia seriata*, and *Botryosphaeria dothidea* of ε-polylysine, chitosan oligomers, their conjugates, *Streptomyces rochei* and *S. lavendofoliae* culture filtrates, and their binary mixtures with chitosan oligomers. In vitro mycelial growth inhibition tests suggest that the efficacy of these treatments, in particular those based on ε-polylysine and ε-polylysine:chitosan oligomers 1:1 *w*/*w* conjugate, against the three Botryosphaeriaceae species would be comparable to or higher than that of conventional synthetic fungicides. In the case of ε-polylysine, EC_90_ values as low as 227, 26.9, and 22.5 µg·mL^−1^ were obtained for *N. parvum*, *D. seriata*, and *B. dothidea*, respectively. Although the efficacy of the conjugate was slightly lower, with EC_90_ values of 507.5, 580.2, and 497.4 µg·mL^−1^, respectively, it may represent a more cost-effective option to the utilization of pure ε-polylysine. The proposed treatments may offer a viable and sustainable alternative for controlling GTDs.

## 1. Introduction

Grapevine trunk diseases (GTDs) have been reported in most grapevine producing regions worldwide, causing a serious decline and loss of productivity. These diseases include black dead arm, caused by *Botryosphaeria dothidea*; esca, which includes vascular symptoms and internal white rot in the trunk; eutypiosis, caused by *Eutypa lata*; Petri disease; black foot; and Phomopsis dieback, being the esca complex the most frequent and increasing syndrome in almost all European countries [1]. A recent International Organization of Vine and Wine (OIV) publication reported that incidence of GTDs was 10% in Spain, 13% in France, and between 8% and 19% in Italy, and that the losses in California were at least 260 M$ per year [2].

A thorough and up-to-date panorama of the state-of-the-art of chemicals (including synthetic organic compounds, inorganic compounds, natural compounds, and plant-defense stimulating compounds) and biocontrol agents that have been tested towards GTDs can be found in the recent review paper by Mondello et al. [2].

Unfortunately, chemical fungicides traditionally used to control aforementioned fungal crop infections, such as sodium arsenite, carbendazim, or tecobunazole, have several drawbacks in terms of toxicity and efficacy, and, in recent years, public pressure to reduce their use has increased. In fact, concerns have been raised about both their environmental impact and the potential associated health risks. In this context, the use of natural antifungals as a feasible alternative is receiving increasing attention.

Among the tested natural compounds, Nascimento et al. [3] reported the antifungal effect of chitosan on several fungal species involved in grapevine decline. Greenhouse experiments using foliar sprays of chitosan on potted grapevine plants growing in a substrate artificially infected with *Phaeomoniella chlamydospora* or *Ilyonectria liriodendri* demonstrated that chitosan significantly improved plant growth and decreased disease incidence. More recently, Cobos et al. [4] reported that chitosan oligosaccharides, garlic extract, and vanillin were able to significantly reduce infection in pruning wounds by *Diplodia seriata*. Galarneau et al. [5] also examined the potential role of antimicrobial phenolic compounds on *Neofusicoccum parvum* and *D. seriata*, two causal fungi of Botryospheria dieback.

ε-polylysine (EPL), a natural antimicrobial produced from aerobic bacterial fermentation by *Streptomyces albulus*, widely used in Japan and USA as an antimicrobial agent in food products, could also be a promising antifungal agent [6]. Although it has been reported to have a strong activity against *Escherichia coli, Staphylococcus aureus*, and *Bacillus subtilis* [7], either alone or in chitosan conjugate compounds, its efficacy has not been assayed against GTDs. 

In a similar fashion, even though beneficial bacteria inhabiting the rhizosphere and/or the endosphere of plants and their secondary metabolites have been put forward by some authors to reduce grapevine pathogen diseases [6], information reported in the literature is limited [8,9,10,11,12]. These biocontrol agents, such as *Streptomyces* spp., would affect pathogen performance by antibiosis, competition for niches and nutrients, interference with pathogen signaling, or by stimulation of host plant defenses.

The aim of the study presented herein has been to assess the in vitro antifungal activity of EPL, EPL:chitosan oligomers (EPL:COS) conjugates, and secondary metabolites from two beneficial actinobacteria (*Streptomyces rochei* and *S. lavendofoliae*) to control *N. parvum*, *D. seriata*, and *B. dothidea*, three of the most frequently isolated fungal pathogens in GTDs. 

## 2. Results

### 2.1. Vibrational Analysis of the ε-polylysine: Chitosan Conjugates

**Figure 1:**
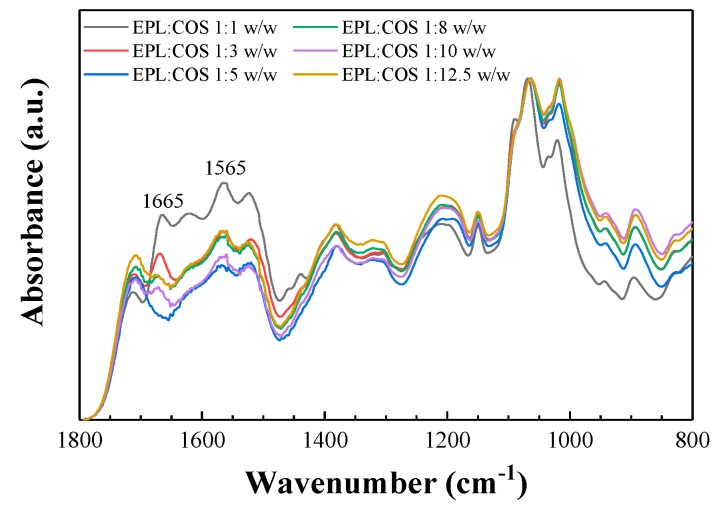
Comparison of the attenuated total reflection (ATR)-Fourier-Transform Infrared (FTIR) spectra of ε-polylysine:chitosan oligomers conjugates prepared with different ε-polylysine:chitosan oligomers mass ratios. Only the fingerprint region is shown.

The vibrational spectra of conjugates prepared with six different EPL:COS mass ratios were examined in order to confirm their secondary structure and to determine the most suitable proportion (Figure 1).

The absorption bands at 1150 cm^−1^ and 1018 cm^−1^ were assigned to asymmetric stretching of the C−O−C bridge and to the skeletal vibration of C−O stretching, respectively [13,14,15]. The absorption band at 895 cm^−1^ could be assigned to the β-D-configuration. There was a shift of amide/amino bands in the reaction products, indicating the progress of Maillard reaction: the absorption peaks at 1659 cm^−1^ and 1597 cm^−1^ (associated with amino groups characteristic of chitosan oligomers) disappeared, and new bands at 1665 cm^−1^ and 1565 cm^−1^ were observed. The appearance of these bands suggest that a Schiff base (C=N bond) was formed between the reducing end of chitosan and the amino groups [16]. Thus, the Fourier-Transform Infrared (FTIR) results showed that ε-polylysine had actually attached to chitosan.

An interesting feature was that the absorbance of the bands associated with Schiff base formation were stronger in the 1:1 EPL:COS conjugate than in the spectra of conjugates prepared with other EPL:COS ratios. Thus, the Schiff base for the 1:1 conjugate seems to feature the desired balance of components to undergo the Amadori rearrangement with formation of ketosamines, but avoiding their subsequent decomposition observed in more COS-rich conjugates. This result was in good agreement with the findings of Liang et al. [7] for EPL:chitosan, who concluded that the conjugate with EPL and chitosan ratio of 1:1 exhibited the strongest antibacterial and antifungal activity. Consequently, the 1:1 EPL:COS conjugate was chosen for the mycelial growth inhibition tests in this study.

### 2.2. Mycelial Growth Inhibition Tests

**Figure 2:**
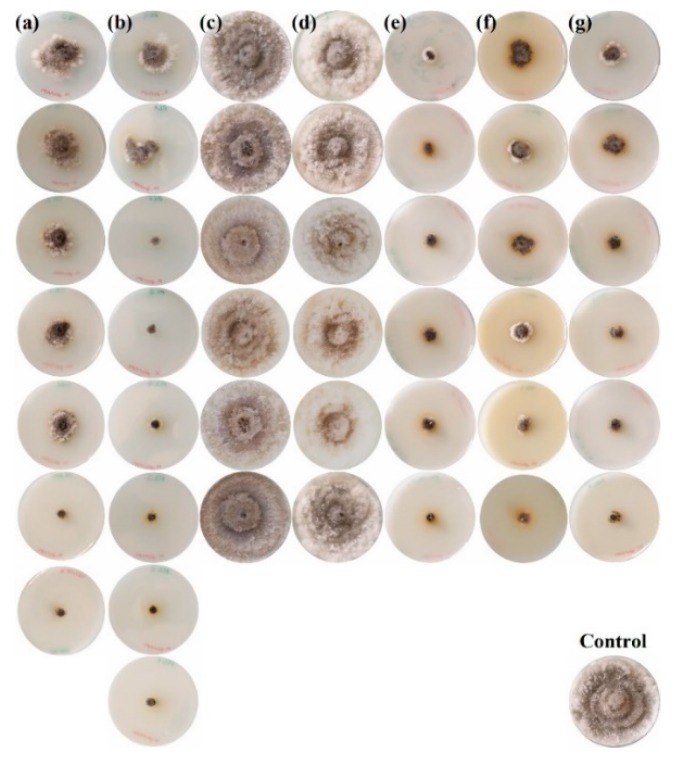
*N. parvum* mycelial growth inhibition assays for: (**a**) chitosan oligomers; (**b**) ε-polylysine; (**c**) *S. rochei* secondary metabolites; (**d**) *S. lavendofoliae* secondary metabolites; (**e**) ε-polylysine:chitosan (1:1 *w*/*w*) conjugates; (**f**) *S. rochei* secondary metabolites + chitosan oligomers (1:1 *w*/*w*); and (**g**) *S. lavendofoliae* secondary metabolites + chitosan oligomers (1:1 *w*/*w*). The concentration of the treatments decreases from top to bottom (doses for each treatment are indicated in Table 3). The petri dish in the bottom right corner shows the PDA control. Only one replicate per each treatment and dose is shown.

**Figure 3:**
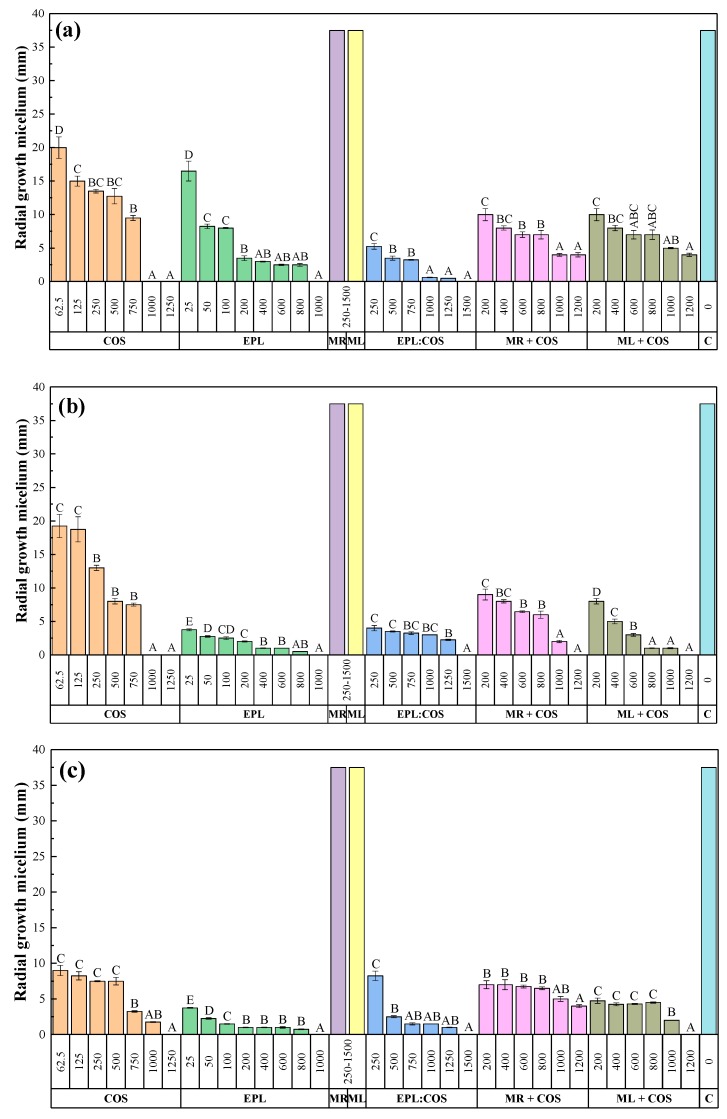
Radial growth values of (**a**) *N. parvum*; (**b**) *D. seriata*; and (**c**) *B. dothidea* in the presence of the different treatments under study at different concentrations (in µg·mL^−1^). COS, EPL, MR, ML and C stand for chitosan oligomers, ε-polylysine, *S. rochei* secondary metabolites, *S. lavendofoliae* secondary metabolites and control, respectively. For MR and ML only one column is shown, since no inhibition was detected at any concentration in the 250–1500 µg·mL^−1^ range. Concentrations labelled with the same uppercase letters are not significantly different at *p* < 0.05 by Tukey’s test. All values are presented as the average of three repetitions. Error bars represent the standard deviation across three replicates.

The in vitro radial growth inhibition attained by each of the treatments against *N. parvum* is depicted in Figure 2, showing only for one replicate per treatment and dose. Those attained against *D. seriata* and *B. dothidea* are depicted in Figures S1 and S2, respectively. The values across the three replicates for the three Botryosphaeriaceae species are summarized in Figure 3.

The increase in the treatment doses resulted in a reduction in the radial growth of the mycelium in all cases, with statistically significant differences amongst the various concentrations (Figure 3), except for the *S. rochei* and *S. lavendofoliae* secondary metabolites-only based treatments (MR and ML, respectively), for which no inhibition was observed.

Doses in the 1000–1500 µg·mL^−1^ range were required to attain full inhibition of the three Botryosphaeriaceae species for the COS, EPL, and EPL:COS conjugate treatments. As regards the activity of MR+COS and ML+COS treatments, differences were observed as a function of the fungal pathogen species. Full inhibition of *D. seriata* was attained for both treatments at a dose of 1200 µg·mL^−1^, whereas it was only observed for ML+COS in the case of *B. dothidea*. MR+COS treatment led to 89% inhibition at the same dose for this latter pathogen. In the case of *N. parvum*, the highest doses of MR+COS and ML+COS led to 83% and 89% inhibition, respectively.

The sensitivity tests results may also be expressed in terms of effective concentrations EC_50_ and EC_90_, that is, the concentrations that reduce mycelial growth by 50% and 90%, respectively (Table 1). Goodness-of-fit analyses revealed good *r*^2^ and low sum of standard errors, showing that parameter fits of sigmoid curves to the dose-response data were significant. In view of the obtained theoretical values, the activity of the treatments—in general terms—would follow the sequence EPL > EPL:COS > ML+COS > COS > MR+COS.

## 3. Discussion

### 3.1. Efficacy of the Treatments

In relation to the efficacy of the composites, although the review paper by Mondello et al. [2] provides a qualitative comparison of different treatment against GTDs, specific inhibition rates with their associated concentrations or effective concentrations were not provided. A survey of such values against the three Botryosphaeriaceae species under study is summarized in Table 2 for comparison purposes.

It may be observed that the EC_50_ values for the treatments presented herein (Table 1), in particular those of EPL and EPL:COS conjugate, were comparable to or better than those of popular synthetic organic compounds used to control GTDs, and only slightly lower than the excellent activities reported for AgNPs.

The results presented for COS were in excellent agreement with those reported by Nascimento et al. [3] and Cobos et al. [4]. However, with regard to this latter study, it should be noted that while the use of polyphenols, such as vanillin or those found in garlic extract, may be suitable against *D. seriata* and other Botryosphaeriaceae strains [17], it may not be advisable against *N. parvum*. Galarneau et al. [5] recently found that *N. parvum* was either uninhibited or promoted by phenolic compounds such as gallic acid, epicatechin, rutin, or piceid. In fact, the authors explained that the ability of *N. parvum* to tolerate these phenolics or utilize them as carbon sources would contribute to its greater virulence compared to *D. seriata*.

The *Streptomyces* spp. secondary metabolites-based treatments showed an unexpected lack of activity when used alone. In fact, the percentage of inhibition of radial growth (PIRG) values, shown in Tables S1–S3, were negative, i.e., the growth of the pathogens was promoted. This was not a case of hormetic response, provided that increasing the concentration did not result in inhibition. The observed mycelial growth promotion may be tentatively ascribed to the presence of molasses and yeast extract in the culture filtrates, together with a poor absorption and bioavailability of the active ingredients in the water-based culture filtrates, resulting from their insolubility or very poor solubility in water. 

In relation to one of the active compounds present in the culture filtrates under test, lankacidin, Harada et al. [26] stated that lankacidin-group antibiotics are scarcely soluble in water and that the parts that dissolved are rapidly decomposed to compounds with no antimicrobial activity. To overcome this problem, they prepared inclusion compounds with cyclodextrins. In this study, this solubility problem was solved by forming polyelectrolyte complexes (PECs) with a polycationic polymer, i.e., chitosan oligomers. These chitosan-based PECs have been reported to exhibit favorable physicochemical properties and to preserve chitosan’s biocompatible characteristics [27], which has made this approach very popular in the drug delivery fields [28,29]. In fact, Zhang et al. [30] previously reported that chitosan behaves as an efficient carrier to deliver streptomycin.

### 3.2. Mechanism of Action

Concerning the mechanism of action (MOA) of the proposed treatments, although the antimicrobial activity of EPL is well documented, its MOA has only been vaguely described. Hyldgaard et al. [31] hypothesized that EPL destabilizes membranes in a carpet-like mechanism by interacting with negatively charged phospholipid head groups, which displace divalent cations and enforce a negative curvature folding on membranes that leads to formation of vesicles/micelles. According to Ye et al. [32], the antimicrobial mechanism of EPL may be attributed not only to disturbances on membrane integrity, but also to oxidative stress by ROS, and to its effects on various gene expressions. 

It is worth noting that the fungicidal activity would likely benefit from the substitution of lysine with arginine residues, provided that previous works have demonstrated the superior cell permeability by arginine polymers over lysine-containing ones [33,34]. Mechanistic evidences indicate that arginine can enhance the activity of both translocating and membrane permeabilizing peptides [35,36]. This would be a potential direction for future studies.

Regarding the inhibition mode of chitosan oligomers, Ing et al. [37] proposed several MOAs. The interaction of chitosan’s positive charge with negatively charged phospholipid components would result in an increased permeability and in leakage of cellular contents. Its chelating action would deprive fungi of trace elements essential for their normal growth. Moreover, its binding to fungal DNA would inhibit mRNA synthesis and affect proteins and enzymes production.

Consequently, the activity of EPL:COS conjugates, as noted by Liang et al. [7], should be referred to an enhanced disruption of their cell membranes, leading to damages of structure, function, and permeability, leakage of intracellular components and the ultimate lysis of the cell.

### 3.3. Applicability to GTDs in vivo

As regards the applicability of the proposed treatments to GTDs in vivo, although it was not covered in this preliminary study, several systems may be envisaged [38]. To reduce symptoms in the field, once the wood is already infected, an approach to be explored would be to apply the products to the soil (injector pole) or to the trunk (trunk injections), mimicking the mechanism activated by winter spraying of sodium arsenite [39]. However, it would be expensive and time-consuming if applied on a large scale [40], and would only be cost-effective when applied in high-value vineyards [41,42].

The proposed antifungal agents may also be administrated by foliar pulverization with minor changes to the formulations (e.g., adding a surfactant as Tween-80). This would be the most practical approach considering the experience of winegrowers. According to Roblin et al. [43], the compounds sprayed on the leaf blades would be able to migrate to the fungal target in the trunk or to trigger the plant defense reaction in distal parts of the plant. In fact, successful use of foliar sprays of chitosan on grapevine plants artificially infested with *Phaeomoniella chlamydospora* or *Neonectria liriodendri* have been reported in the literature [3]. However, this application method has the major drawback that the treatments may be easily washed off by rainfall [44]. If this approach was to be chosen, sprays after the period of vintage should be useful since, at this period, the phloem sap begins to be directed in a descending flow towards the roots, assuring the transport of the compounds towards the fungi [43].

Alternatively, as a preventive measure, the active ingredients may also be used to protect pruning wounds to avoid grapevine infection and to limit fungal expansion in the plant, either as painted pastes or as liquid formulations. This application method was evaluated against *D. seriata* and *P. chlamydospora* in field trials by Cobos et al. [4], using chitosan oligosaccharides, vanillin, and garlic extract, and resulted in a significant decrease in plant mortality and in the infection rate. Nonetheless, to improve the adherence of the treatments, thickener agents would need to be added to the formulations: e.g., starches, vegetable gums, pectin, or clays such as halloysite.

### 3.4. Significance of the Reported Findings

Although follow-up studies involving in vivo assays and field tests would be necessary to draw firm conclusions on the effectiveness of the application of the proposed treatments, the fact that they reached higher mycelial growth inhibition than that of commercial fungicides makes them promising candidates for the effective control of botryosphaeriaceous diseases. 

It is also worth noting that the three fungal species tested in the present study are not only pathogens of grapevine, but also of other commercially important woody plants. For instance, *D. seriata* and *B. dothidea* are phytopathogens of apple [22], *N. parvum* causes dieback in avocado [45], *B. dothidea* causes branch dieback of olive [46], and the three of them are associated with branch cankers on almond trees [47]. Consequently, the results of this study may also find application in other pathosystems, resulting in an even higher ecological and economic impact.

## 4. Materials and Methods

### 4.1. Reagents, Bacteria and Fungi

High molecular weight chitosan (CAS 9012-76-4; 310000-375000 Da) was purchased from Hangzhou Simit Chemical Technology Co., Ltd. (Hangzhou, China). ε-polylysine (CAS 25104-18-1), phosphate buffer (for microbiology, APHA, pH 7.2), ethyl acetate (CAS 141-78-6; ≥99.5%), and citric acid (CAS 77-92-9; ≥99.5%) were supplied by Sigma-Aldrich Química S.A. (Madrid, Spain). Neutrase^®^ 0.8L enzyme was supplied by Novozymes (Bagsvaerd, Denmark). Potato dextrose agar (PDA), yeast extract, and Bacto^TM^ Peptone were purchased from Becton, Dickinson and Company (Franklin Lakes, NJ, USA). Starch casein agar (SCA), Mueller Hinton agar, and malt extract agar (MEA) came from Oxoid Ltd. (Hampshire, UK). Molasses were supplied by ACOR, Sociedad Cooperativa General Agropecuaria (Castilla y León, España).

The three fungal isolates under study, viz. *Diplodia seriata* (ITACYL_F079), *Neofusicoccum parvum* (ITACYL_F111), and *Botryosphaeria dothidea* (ITACYL_F141), were supplied by ITACYL, Instituto Tecnológico Agrario de Castilla y León (Castilla y León, España).

The two *Streptomyces* spp. strains from which secondary metabolites were produced, *Streptomyces lavendofoliae* (DSM 40217) and *Streptomyces rochei* (DSM 41729) were purchased from DSMZ (Deutsche Sammlung von Mikroorganismen und Zellkulturen; Braunschweig, Germany).

### 4.2. Equipment

A probe-type UIP1000hdT ultrasonicator (Hielscher, Teltow, Germany; 1000 W, 20 kHz) was used for solutions sonication.

To incubate the flasks, controlling the temperature and the stirring speed, an ECOLAN 60 (Labolan; Esparza de Galar, Navarra, Spain) orbital stirrer incubator was used.

Functional groups were identified by Fourier-Transform Infrared spectroscopy with a Nicolet iS50 (Thermo Scientific, Waltham, MA, USA) apparatus equipped with a diamond attenuated total reflection (ATR) module. The spectra were collected in the 400–4000 cm^−1^ region with a 1 cm^−1^ spectral resolution; 64 scans were co-added and the resulting interferogram was averaged. The ATR-FTIR spectra were corrected using the advanced ATR correction algorithm [47] available in OMNIC^TM^ software suite.

### 4.3. Preparation of Chitosan Oligomers

Chitosan oligomers were obtained according to the enzymatic procedure described by Santos-Moriano et al. [48], with slight modifications. 20 g of high molecular weight chitosan were dissolved in 1000 mL of Milli-Q water by adding citric acid under constant stirring at 60 °C. Once dissolved, Neutrase^®^ 0.8 L (1.67 g·L^−1^) was added in order to degrade the polymer chains. The mixture was sonicated for 3 min in cycles of 1 min with sonication and 1 min without sonication to keep the temperature in the 30–60 °C range [14]. At the end of the process, a solution with a pH in the four to six interval with oligomers of molecular weight < 2000 Da was obtained.

### 4.4. ε-polylysine Treatment

For the preparation of the ε-polylysine treatment, 2 g of EPL were dissolved in 1000 mL of Milli-Q water. The mixture was sonicated for 3 min in cycles of 1 min with sonication and 1 min without sonication so that the temperature remained in the 30–60 °C range. 

### 4.5. Synthesis of ε-polylysine: Chitosan Oligomers Conjugates

Conjugated complexes of ε-polylysine and chitosan oligomers were prepared at different mass ratios, namely 1:1, 1:3, 1:5 1:8, 1:10, and 1:12.5 *w*/*w*, respectively. The appropriate amounts of each component were dissolved in Milli-Q water using sonication (5 cycles of 5 min/cycle, taking care not to exceed 60 °C). The resulting solutions were lyophilized, and then heated at 60 °C under 60% relative humidity for 24 h. This synthesis procedure was analogous to other procedures described in the literature for the preparation of EPL:COS conjugates through Maillard reaction [7,49,50]. Only the conjugate with the highest expected activity was assayed in the mycelial growth inhibition tests.

### 4.6. Secondary Metabolites Production from Streptomyces spp. Strains

Two strains of the genus *Streptomyces*, viz. *Streptomyces lavendofoliae* DSM 40217 and *Streptomyces rochei* DSM 41729 were seeded on starch casein agar medium plates at 28 °C for 10 days. The plates were stored at 4 °C. For long-term storage, lyophilizates from both strains were used.

In order to obtain the secondary metabolites, the method described by Sadigh-Eteghad et al. [51] was followed. Once the fermentation was complete, each final solution of the cultures of both strains was treated with 50 mL of phosphate buffer (pH 6.4) and was sonicated for 5 min. The solutions were then filtered through sterile muslin cloth twice. These solutions (culture filtrates) were used for the mycelial growth inhibition tests.

In order to determine the concentration of bioactive compounds in aforementioned solutions (and the doses used in the inhibition tests), the filtrates were centrifuged, and the supernatant was extracted with 100 mL of ethyl acetate. The solvent with the crude bioactive compounds was concentrated under reduced pressure and then lyophilized. The culture filtrates had a concentration of approx. 2000 μg∙mL^−1^ (1958 μg∙mL^−1^ for *S. lavendofoliae* secondary metabolites and 1877 μg∙mL^−1^ for *S. rochei* secondary metabolites), in agreement with Pazhanimurugan et al. [52]. The bioactive compounds in the secondary metabolites of *S. lavendofoliae* and *S. rochei* are summarized in Table S4. 

### 4.7. Synthesis of Chitosan Oligomers-secondary Metabolites Inclusion Compounds

Secondary metabolites, either from *S. lavendofoliae* or from *S. rochei*, and chitosan oligomers mixtures were prepared by mixing in 1:1 (*w*/*w*) ratio of their respective solutions (2000 μg∙mL^−1^ of bioactive compounds + 2000 μg∙mL^−1^ COS), followed by sonication. The resulting solutions (ML+COS and MR+COS) were assayed at different concentrations in the inhibition tests.

### 4.8. In vitro Mycelial Growth Inhibition Tests

The biological activity of the treatments under study was determined by the agar dilution method: aliquots of the original solutions of the various treatments, obtained by dilution of the respective “mother” solutions, were incorporated into the PDA medium to obtain the final concentrations indicated in Table 3. It should be clarified that the tested concentrations were not the same all treatments due to difficulties associated with the estimation of the molecular weights of the polymeric reagents from their viscosities. Petri dishes containing only PDA culture medium (20 mL) were used as the control.

The mycelial discs of pathogen (5 mm in diameter) were removed from the margins of 7-day-old PDA cultures and transferred to the petri dishes (in triplicate). Plates were incubated at 25 °C. The measurements of fungal growth for *D. seriata* and *N. parvum* were taken two, four and five days after inoculation. In contrast, for *B. dothidea*, measurements were carried out two, four and six days after inoculation, provided that mycelial growth was slower for this later fungus in the control plates.

The inhibition of mycelial growth, or the efficacy of the compound analyzed, for each treatment and concentration, was calculated by the formula:

Percentage inhibition of radial mycelium growth (%) = ((R_1_ − R_2_)/R_1_) × 100 (1)

where R_1_ and R_2_ correspond to the average radial growth of the fungal mycelium in the control medium (pure PDA) and in the fungicide-amended medium, respectively.

The results were also expressed as the effective concentrations that reduced mycelial growth by 50% and 90% (EC_50_ and EC_90_, respectively), which were determined by the regression of the radial growth inhibition values (%) against the log_10_ values of the concentrations of antifungal compounds using PROBIT in IBM SPSS Statistics v.25 software. This regression procedure fits the dose-response curve to a sigmoid and calculates the values, with 95% CI, of the dose variable that correspond to a series of probabilities.

### 4.9. Statistical Analyses

Data were subjected to analysis of variance (ANOVA) in IBM SPSS Statistics v.25 software. Tukey’s HSD test at 0.05 probability level (*p* < 0.05) was used for the *post hoc* comparison of means.

## 5. Conclusions

The efficacy of ε-polylysine, chitosan oligomers, ε-polylysine:chitosan oligomers conjugates, two *Streptomyces* spp. secondary metabolites, and the combinations of the latter two with chitosan oligomers were examined in vitro against *N. parvum*, *D. seriata* and *B. dothidea*. On the basis of vibrational spectroscopy data, a 1:1 *w*/*w* mass ratio was chosen for the EPL:COS conjugate, for which an optimum Schiff base was formed. From the mycelial growth inhibition tests it was found that, in spite of the remarkable contents in bioactive compounds in the culture filtrates, the secondary metabolites of *S. rochei* and *S. lavendofoliae* did not inhibit any of the GTD-related fungi, probably due to hydrophobicity reasons. In contrast, upon formation of polyelectrolyte complexes with chitosan oligomers, inhibitions above 80% were attained. In view of the calculated effective concentration values, the antifungal activity of the treatments would follow the sequence EPL > EPL:COS > ML+COS > COS > MR+COS. EC_50_ values below 100 µg·mL^−1^ were obtained for all the assayed treatments, suggesting that they could be a viable alternative to conventional synthetic fungicides. In particular, ε-polylysine and ε-polylysine:chitosan oligomers may be put forward as the most promising options, due to the high efficacy of the former and the trade-off between efficacy and cost associated with the latter. In the current context in which the use of synthetic chemical pesticides is more and more restricted, this work constitutes a necessary step for developing efficient treatments that take into account the importance of environmental protection within the scope of sustainable development.

## Figures and Tables

**Table 1:** Effective concentrations that inhibited mycelial growth by 50% and 90% (EC_50_ and EC_90_, respectively).

Pathogen	Concentration (µg·mL^−1^)	Treatment				
		COS	EPL	EPL:COS	MR + COS	ML + COS
*N. parvum*	EC_50_	60.7	16.0	11.2	67.2	46.7
	EC_90_	1270.0	227.0	507.5	2074.2	1101.7
*D. seriata*	EC_50_	94.3	0.3	11.6	45.1	30.7
	EC_90_	1120.7	26.9	580.2	906.9	498.2
*B. dothidea*	EC_50_	1.8	0.4	4.2	15.8	10.7
	EC_90_	689.5	22.5	497.4	1019.0	490.3

**Table 2:** Concentration values and associated inhibition rates, or EC_50_ values, reported in the literature for other active compounds against the three Botryosphaeriaceae species under study.

Fungicide	Fungal Species	Concentration (µg·mL^−1^)	Inhibition rate (%)	EC_50_(µg·mL^−1^)	Ref.
Tebuconazole	*N. parvum*			90	[18]
	*D. seriata*			150	
Pyraclostrobin	*N. parvum*			100	
	*D. seriata*			250	
Carbendazim, tebuconazole, iprodione, fludioxonil, fluazinam, flusilazole, penconazole, procymidone, myclobutanil, pyraclostrobin	*N. parvum*			360–440 *	[19]
	*D. seriata*			530–620 *	
	*B. dothidea*			450 *	
Carbendazim	*N. parvum*			40	[20]
Tebuconazole				130	
Iprodione				750	
Tecobunazole	*D. seriata*			300	[21]
Fe NPs (FeNPs + neem leaf extract)	*D. seriata*	100 (FeNPs / FeNPs+neem 1:1)	79/80.3		[22]
	*B. dothidea*		83/82.5		
AgNPs	*N. parvum*	40	84		[23]
AgNPs		30	81		[24]
Lemon essential oil (limonene, neral, β-pinene, and γ-terpinene) in DMSO	*B. dothidea*	2500	48.1		[25]
Chitosan oligosaccharin (mol. wt. <3 kDa)	*Botryosphaeria* sp.			1.56	[3]
Chitosan oligosaccharides	*D. seriata*	1000	100		[4]
Vanillin		1000	89.8		
Garlic extract		40000	75.3		

* Data pooled across fungicides to provide mean EC_50_ values for isolate sensitivity in the original study.

**Table 3:** Concentrations assayed for each of the treatments in the mycelial growth inhibition tests. COS, PL, MR and ML stand for chitosan oligomers, ε-polylysine, *S. rochei* secondary metabolites, and *S. lavendofoliae* secondary metabolites, respectively.

Treatment	Concentrations Assayed in the Mycelial Growth Inhibition Tests (μg∙mL^−1^)
COS	62.5, 125, 250, 500, 750, 1000, 1250, 1500
EPL	25, 50, 100, 200, 400, 600, 800, 1000
MR	250, 500, 750, 1000, 1250, 1500
ML	250, 500, 750, 1000, 1250, 1500
EPL:COS	250, 500, 750, 1000, 1250, 1500
MR+COS	200, 400, 600, 800, 1000, 1200
ML+COS	200, 400, 600, 800, 1000, 1200

## Supplementary Materials

The following are available online at https://www.mdpi.com/2079-6382/8/3/99/s1, Figure S1: *D. seriata* mycelial growth inhibition assays, Figure S2: *B. dothidea* mycelial growth inhibition assays, Table S1: Radial growth of mycelium (RG) and percentage of inhibition of radial growth (PIRG) of the different treatments against *N. parvum* two, four and five days after inoculation, Table S2: Radial growth of mycelium (RG) and percentage of inhibition of radial growth (PIRG) of the different treatments against *D. seriata* two, four and five days after inoculation, Table S3: Radial growth of mycelium (RG) and percentage of inhibition of radial growth (PIRG) of the different treatments against *B. dothidea* two, four and six days after inoculation, Table S4: Bioactive secondary metabolites produced by *S. lavendofoliae* and *S. rochei*.
