# Supplementary material for: Antifungal Agents Based on Chitosan Oligomers, ε-polylysine and Streptomyces spp. Secondary Metabolites against Three Botryosphaeriaceae Species
Source: Antibiotics (Basel). 2019 Jul 20;8(3):99. doi: 10.3390/antibiotics8030099 (PMC6783921; doi:10.3390/antibiotics8030099)
Supplement: Supplementary file 1 [file antibiotics-08-00099-s001.pdf]

Antifungal Agents Based on Chitosan Oligomers, ε-polylysine and *Streptomyces* spp. Secondary Metabolites Against Three Botryosphaeriaceae Species

Laura Buzón-Durán, Jesús Martín-Gil, Eduardo Pérez-Lebeña, David Ruano-Rosa, José L. Revuelta, José Casanova-Gascón, M. Carmen Ramos-Sánchez and Pablo Martín-Ramos*

Supplementary Materials

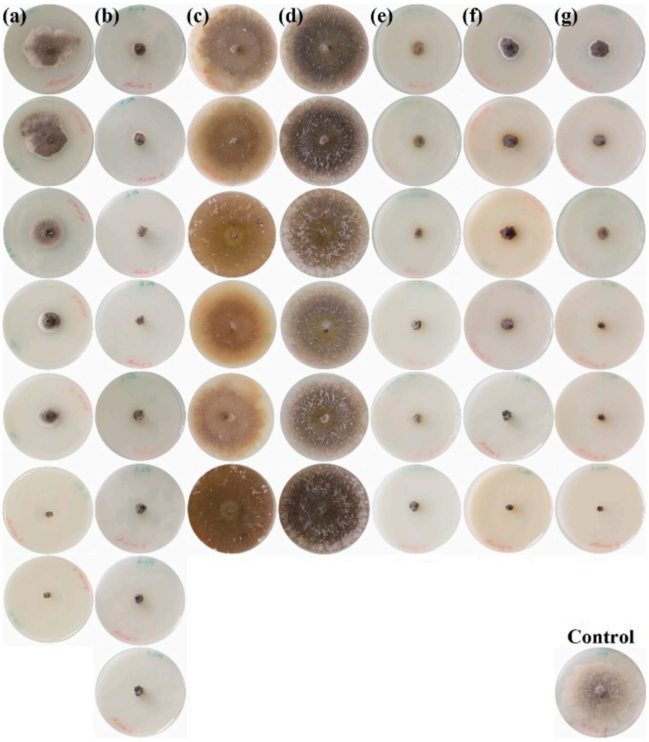

Figure S1. *D. seriata* mycelial growth inhibition assays for: (**a**) chitosan oligomers; (**b**) ε-polylysine; (**c**) *S. rochei* secondary metabolites; (**d**) *S. lavendofoliae* secondary metabolites; (**e**) ε-polylysine:chitosan (1:1 w/w) conjugates; (**f**) *S. rochei* secondary metabolites + chitosan oligomers (1:1 w/w); (**g**) *S. lavendofoliae* secondary metabolites + chitosan oligomers (1:1 w/w). The concentration of the treatments decreases from top to bottom (doses for each treatment are indicated in **Error! Reference source not found.**). The Petri dish in the bottom right corner shows the PDA control. Only one replicate per treatment and dose is shown.

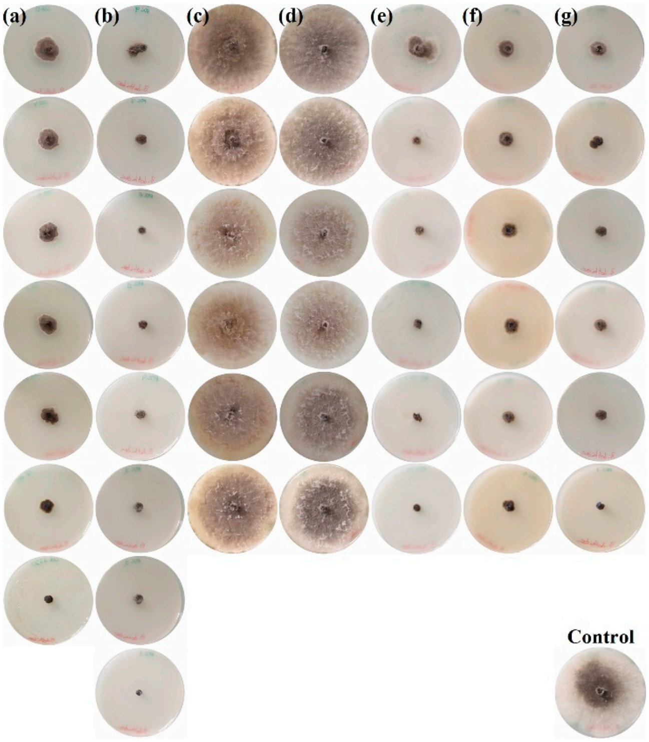

Figure S2. *B. dothidea* mycelial growth inhibition assays for: (**a**) chitosan oligomers; (**b**) ε-polylysine; (**c**) *S. rochei* secondary metabolites; (**d**) *S. lavendofoliae* secondary metabolites; (**e**) ε-polylysine:chitosan (1:1 w/w) conjugates; (**f**) *S. rochei* secondary metabolites + chitosan oligomers (1:1 w/w); (**g**) *S. lavendofoliae* secondary metabolites + chitosan oligomers (1:1 w/w). The concentration of the treatments decreases from top to bottom (doses for each treatment are indicated in **Error! Reference source not found.**). The Petri dish in the bottom right corner shows the PDA control. Only one replicates per treatment and dose is shown.

Table S1. Radial growth of mycelium (RG) and percentage of inhibition of radial growth (PIRG) of the different treatments against *N. parvum* 2, 4 and 5 days after inoculation. Means followed by the same letter are not significantly different according to Tukey's HSD test ($p < 0.05$).

**Treatment**	**Dose (μg·mL⁻¹)**	***N. parvum***					
		**2 days**		**4 days**		**5 days**	
		**RG (mm)**	**PIRG (%)**	**RG (mm)**	**PIRG (%)**	**RG (mm)**	**PIRG (%)**
Control	-	18 ± 1.62	-	29.5 ± 2.65	-	38 ± 3.42	-
COS	62.5	9.25 ± 0.56 d	48.6	17.00 ± 1.19 c	42.4	20.00 ± 1.60 d	46.7
	125	5.75 ± 0.23 c	68.1	8.50 ± 0.68 b	71.2	15.00 ± 0.75 c	60.0
	250	4.00 ± 0.36 b	77.8	6.50 ± 0.59 b	78.0	13.50 ± 0.27 bc	64.0
	500	4.50 ± 0.27 bc	75.0	5.50 ± 0.50 b	81.4	12.75 ± 1.15 bc	66.0
	750	4.75 ± 0.29 bc	73.6	7.00 ± 0.56 b	76.3	9.50 ± 0.38 b	74.7
	1000	0.00 ± 0.00 a	100.0	0.00 ± 0.00 a	100.0	0.00 ± 0.00 a	100.0
	1250	0.00 ± 0.00 a	100.0	0.00 ± 0.00 a	100.0	0.00 ± 0.00 a	100.0
	1500	0.00 ± 0.00 a	100.0	0.00 ± 0.00 a	100.0	0.00 ± 0.00 a	100.0
EPL	25	3.25 ± 0.29 d	81.9	13.75 ± 0.69 e	53.4	16.50 ± 1.49 d	56.0
	50	3.50 ± 0.32 d	80.6	4.75 ± 0.38 d	83.9	8.25 ± 0.33 c	78.0
	100	1.75 ± 0.16 c	90.3	2.25 ± 0.23 bc	92.4	8.00 ± 0.08 c	78.7
	200	1.30 ± 0.10 bc	92.8	3.50 ± 0.18 cd	88.1	3.50 ± 0.35 b	90.7
	400	1.25 ± 0.08 bc	93.1	2.00 ± 0.12 bc	93.2	3.00 ± 0.06 ab	92.0
	600	1.00 ± 0.04 bc	94.4	2.00 ± 0.00 bc	93.2	2.50 ± 0.10 ab	93.3
	800	0.75 ± 0.01 ab	95.8	1.50 ± 0.03 ab	94.9	2.50 ± 0.23 ab	93.3
	1000	0.00 ± 0.00 a	100.0	0.00 ± 0.00 a	100.0	0.00 ± 0.00 a	100.0
MR	250	28.00 ± 2.24 a	−55.6	37.00 ± 1.85 b	−25.4	37.50 ± 0.00 a	0.0
	500	26.00 ± 1.56 a	−44.4	34.00 ± 1.70 ab	−15.3	37.50 ± 0.00 a	0.0
	750	26.00 ± 0.52 a	−44.4	32.00 ± 0.96 ab	−8.5	37.50 ± 0.00 a	0.0
	1000	25.00 ± 2.50 a	−38.9	30.00 ± 1.50 ab	−1.7	37.50 ± 0.00 a	0.0
	1250	25.00 ± 2.50 a	−38.9	29.00 ± 1.74 ab	1.7	37.50 ± 0.00 a	0.0
	1500	25.00 ± 0.25 a	−38.9	28.00 ± 1.40 a	5.1	37.50 ± 0.00 a	0.0
ML	250	28.00 ± 2.80 a	−55.6	37.00 ± 3.70 a	−25.4	37.50 ± 0.00 a	0.0
	500	25.00 ± 2.25 a	−38.9	36.00 ± 2.16 a	−22.0	37.50 ± 0.00 a	0.0
	750	24.00 ± 1.20 a	−33.3	33.00 ± 0.33 a	−11.9	37.50 ± 0.00 a	0.0
	1000	23.00 ± 1.61 a	−27.8	32.00 ± 0.96 a	−8.5	37.50 ± 0.00 a	0.0
	1250	23.00 ± 1.38 a	−27.8	31.00 ± 2.48 a	−5.1	37.50 ± 0.00 a	0.0
	1500	23.00 ± 0.92 a	−27.8	30.00 ± 0.60 a	−1.7	37.50 ± 0.00 a	0.0
EPL:COS	250	1.00 ± 0.10 c	94.4	3.50 ± 0.35 c	88.1	5.25 ± 0.42 c	86.0
	500	1.00 ± 0.09 c	94.4	2.50 ± 0.23 b	91.5	3.50 ± 0.32 b	90.7
	750	0.50 ± 0.01 b	97.2	1.75 ± 0.04 b	94.1	3.25 ± 0.07 b	91.3
	1000	0.00 ± 0.00 a	100.0	0.50 ± 0.05 a	98.3	0.63 ± 0.06 a	98.3
	1250	0.00 ± 0.00 a	100.0	0.75 ± 0.02 a	97.5	0.50 ± 0.01 a	98.7
	1500	0.00 ± 0.00 a	100.0	0.00 ± 0.00 a	100.0	0.00 ± 0.00 a	100.0
MR+COS	200	5.75 ± 0.23 d	68.1	8.00 ± 0.80 d	72.9	10.00 ± 0.90 c	73.3
	400	5.25 ± 0.37 d	70.8	6.00 ± 0.24 c	79.7	8.00 ± 0.32 bc	78.7
	600	3.00 ± 0.27 c	83.3	5.00 ± 0.15 bc	83.1	7.00 ± 0.42 b	81.3
	800	1.75 ± 0.11 b	90.3	4.00 ± 0.16 b	86.4	7.00 ± 0.63 b	81.3
	1000	1.00 ± 0.05 ab	94.4	1.00 ± 0.05 a	96.6	4.00 ± 0.20 a	89.3
	1200	0.00 ± 0.00 a	100.0	0.00 ± 0.00 a	100.0	4.00 ± 0.32 a	89.3
ML+COS	200	5.75 ± 0.58 d	68.1	6.00 ± 0.60 c	79.7	10.00 ± 0.90 c	73.3
	400	5.25 ± 0.37 d	70.8	6.00 ± 0.48 c	79.7	8.00 ± 0.40 bc	78.7
	600	3.00 ± 0.12 c	83.3	5.00 ± 0.45 bc	83.1	7.00 ± 0.63 abc	81.3
	800	1.75 ± 0.04 bc	90.3	3.00 ± 0.21 ab	89.8	7.00 ± 0.70 abc	81.3
	1000	1.00 ± 0.09 ab	94.4	2.00 ± 0.14 a	93.2	5.00 ± 0.10 ab	86.7
	1200	0.00 ± 0.00 a	100.0	1.00 ± 0.01 a	96.6	4.00 ± 0.24 a	89.3

COS, EPL, MR and ML stand for chitosan oligomers, ε-polylysine, *S. rochei* secondary metabolites and *S. lavendofoliae* secondary metabolites, respectively.

Table S2. Radial growth of mycelium (RG) and percentage of inhibition of radial growth (PIRG) of the different treatments against *D. seriata* 2, 4 and 5 days after inoculation. Means followed by the same letter are not significantly different according to Tukey's HSD test ($p < 0.05$).

Treatment	Dose (μg·mL⁻¹)	*D. seriata*					
		2 days		4 days		5 days	
		RG (mm)	PIRG (%)	RG (mm)	PIRG (%)	RG (mm)	PIRG (%)
Control	-	23 ± 0.69	-	28 ± 1.96	-	38 ± 2.95	-
COS	62.5	19.25 ± 1.35 d	16.3	15.25 ± 0.31 d	45.5	19.25 ± 1.73 c	48.7
	125	18.75 ± 0.38 d	18.5	14.00 ± 0.42 d	50.0	18.75 ± 1.88 c	50.0
	250	13.00 ± 1.30 c	43.5	12.00 ± 0.00 c	57.1	13.00 ± 0.39 b	65.3
	500	8.00 ± 0.64 b	65.2	5.75 ± 0.35 b	79.5	8.00 ± 0.40 b	78.7
	750	7.50 ± 0.53 b	67.4	5.50 ± 0.22 b	80.4	7.50 ± 0.23 b	80.0
	1000	0.00 ± 0.00 a	100.0	0.00 ± 0.00 a	100.0	0.00 ± 0.00 a	100.0
	1250	0.00 ± 0.00 a	100.0	0.00 ± 0.00 a	100.0	0.00 ± 0.00 a	100.0
	1500	0.00 ± 0.00 a	100.0	0.00 ± 0.00 a	100.0	0.00 ± 0.00 a	100.0
EPL	25	3.00 ± 0.24 d	87.0	3.50 ± 0.18 f	87.5	3.75 ± 0.15 e	90.0
	50	1.50 ± 0.09 c	93.5	2.25 ± 0.09 e	92.0	2.75 ± 0.11 d	92.7
	100	1.00 ± 0.09 bc	95.7	1.00 ± 0.08 d	96.4	2.50 ± 0.20 cd	93.3
	200	1.00 ± 0.10 bc	95.7	1.50 ± 0.11 c	94.6	2.00 ± 0.08 c	94.7
	400	0.50 ± 0.04 ab	97.8	1.00 ± 0.07 c	96.4	1.00 ± 0.04 b	97.3
	600	0.00 ± 0.00 a	100.0	0.50 ± 0.02 b	98.2	1.00 ± 0.01 b	97.3
	800	0.00 ± 0.00 a	100.0	0.00 ± 0.00 a	100.0	0.50 ± 0.02 ab	98.7
	1000	0.00 ± 0.00 a	100.0	0.00 ± 0.00 a	100.0	0.00 ± 0.00 a	100.0
MR	250	35.00 ± 1.75 a	−52.2	37.50 ± 0.00 a	−33.9	37.50 ± 0.00 a	0.0
	500	33.00 ± 1.32 a	−43.5	37.50 ± 0.00 a	−33.9	37.50 ± 0.00 a	0.0
	750	32.00 ± 1.92 a	−39.1	36.00 ± 2.88 a	−28.6	37.50 ± 0.00 a	0.0
	1000	30.00 ± 2.70 a	−30.4	35.00 ± 0.00 a	−25.0	37.50 ± 0.00 a	0.0
	1250	29.00 ± 1.74 a	−26.1	35.00 ± 2.10 a	−25.0	37.50 ± 0.00 a	0.0
	1500	29.00 ± 1.45 a	−26.1	35.00 ± 2.10 a	−25.0	37.50 ± 0.00 a	0.0
ML	250	35.00 ± 0.35 a	−52.2	37.50 ± 0.00 a	−33.9	37.50 ± 0.00 a	0.0
	500	33.00 ± 0.33 a	−43.5	37.50 ± 0.00 a	−33.9	37.50 ± 0.00 a	0.0
	750	33.00 ± 1.98 a	−43.5	36.00 ± 0.72 a	−28.6	37.50 ± 0.00 a	0.0
	1000	31.00 ± 0.62 a	−34.8	35.00 ± 2.80 a	−25.0	37.50 ± 0.00 a	0.0
	1250	30.00 ± 2.70 a	−30.4	35.00 ± 1.75 a	−25.0	37.50 ± 0.00 a	0.0
	1500	28.50 ± 0.57 a	−23.9	35.00 ± 2.10 a	−25.0	37.50 ± 0.00 a	0.0
EPL:COS	250	2.50 ± 0.18 d	89.1	3.50 ± 0.32 d	87.5	4.00 ± 0.40 c	89.3
	500	2.25 ± 0.14 d	90.2	2.50 ± 0.20 c	91.1	3.50 ± 0.07 c	90.7
	750	1.50 ± 0.14 c	93.5	1.75 ± 0.09 bc	93.8	3.25 ± 0.20 bc	91.3
	1000	1.00 ± 0.04 bc	95.7	1.50 ± 0.03 b	94.6	3.00 ± 0.03 bc	92.0
	1250	0.50 ± 0.01 ab	97.8	1.00 ± 0.07 b	96.4	2.25 ± 0.09 b	94.0
	1500	0.00 ± 0.00 a	100.0	0.00 ± 0.00 a	100.0	0.00 ± 0.00 a	100.0
MR+COS	200	5.00 ± 0.25 c	78.3	7.00 ± 0.70 b	75.0	9.00 ± 0.81 c	76.0
	400	5.00 ± 0.15 c	78.3	6.00 ± 0.60 b	78.6	8.00 ± 0.24 bc	78.7
	600	3.00 ± 0.03 b	87.0	5.00 ± 0.05 b	82.1	6.45 ± 0.13 b	82.8
	800	3.00 ± 0.27 b	87.0	5.00 ± 0.05 b	82.1	6.00 ± 0.54 b	84.0
	1000	2.00 ± 0.18 b	91.3	2.00 ± 0.14 a	92.9	2.00 ± 0.18 a	94.7
	1200	0.00 ± 0.00 a	100.0	0.00 ± 0.00 a	100.0	0.00 ± 0.00 a	100.0
ML+COS	200	6.00 ± 0.54 d	73.9	6.00 ± 0.54 c	78.6	8.00 ± 0.40 d	78.7
	400	3.00 ± 0.24 c	87.0	5.00 ± 0.45 c	82.1	5.00 ± 0.35 c	86.7
	600	2.00 ± 0.02 bc	91.3	3.00 ± 0.12 b	89.3	3.00 ± 0.21 b	92.0
	800	1.00 ± 0.07 ab	95.7	1.00 ± 0.04 a	96.4	1.00 ± 0.03 a	97.3
	1000	0.50 ± 0.02 a	97.8	1.00 ± 0.08 a	96.4	1.00 ± 0.09 a	97.3
	1200	0.00 ± 0.00 a	100.0	0.00 ± 0.00 a	100.0	0.00 ± 0.00 a	100.0

COS, EPL, MR and ML stand for chitosan oligomers, ε-polylysine, *S. rochei* secondary metabolites and *S. lavendofoliae* secondary metabolites, respectively.

Table S3. Radial growth of mycelium (RG) and percentage of inhibition of radial growth (PIRG) of the different treatments against *B. dothidea* 2, 4 and 6 days after inoculation. Means followed by the same letter are not significantly different according to Tukey's HSD test ($p < 0.05$).

**Treatment**	**Dose ($\mu g \cdot mL^{-1}$)**	***B. dothidea***					
		**2 days**		**4 days**		**5 days**	
		**RG (mm)**	**PIRG (%)**	**RG (mm)**	**PIRG (%)**	**RG (mm)**	**PIRG (%)**
Control	-	19 ± 0.57	-	33 ± 1.65	-	38 ± 2.66	-
COS	62.5	5.00 ± 0.45 b	73.7	6.50 ± 0.33 c	80.3	9.00 ± 0.72 c	76.0
	125	5.50 ± 0.22 b	71.1	6.00 ± 0.60 c	81.8	8.25 ± 0.58 c	78.0
	250	5.00 ± 0.45 b	73.7	5.50 ± 0.55 c	83.3	7.50 ± 0.08 c	80.0
	500	4.75 ± 0.38 b	75.0	5.50 ± 0.50 c	83.3	7.50 ± 0.53 c	80.0
	750	1.50 ± 0.08 a	92.1	2.50 ± 0.08 b	92.4	3.25 ± 0.10 b	91.3
	1000	0.25 ± 0.02 a	98.7	1.25 ± 0.04 ab	96.2	1.75 ± 0.07 ab	95.3
	1250	0.00 ± 0.00 a	100.0	0.00 ± 0.00 a	100.0	0.00 ± 0.00 a	100.0
	1500	0.00 ± 0.00 a	100.0	0.00 ± 0.00 a	100.0	0.00 ± 0.00 a	100.0
EPL	25	1.75 ± 0.07 e	90.8	3.25 ± 0.13 f	90.2	3.75 ± 0.08 e	90.0
	50	1.25 ± 0.03 d	93.4	2.25 ± 0.05 e	93.2	2.25 ± 0.11 d	94.0
	100	0.50 ± 0.01 c	97.4	1.00 ± 0.10 d	97.0	1.50 ± 0.03 c	96.0
	200	0.25 ± 0.02 b	98.7	0.50 ± 0.04 bc	98.5	1.00 ± 0.04 b	97.3
	400	0.00 ± 0.00 b	100.0	0.50 ± 0.03 bc	98.5	1.00 ± 0.03 b	97.3
	600	0.25 ± 0.01 a	98.7	0.75 ± 0.03 cd	97.7	1.00 ± 0.10 b	97.3
	800	0.00 ± 0.00 a	100.0	0.25 ± 0.03 ab	99.2	0.75 ± 0.07 b	98.0
	1000	0.00 ± 0.00 a	100.0	0.00 ± 0.00 a	100.0	0.00 ± 0.00 a	100.0
MR	250	29.00 ± 1.16 a	−52.6	35.00 ± 1.05 a	−6.1	37.50 ± 0.00 a	0.0
	500	27.00 ± 1.35 a	−42.1	33.00 ± 2.64 a	0.0	37.50 ± 0.00 a	0.0
	750	26.00 ± 1.30 a	−36.8	31.00 ± 1.24 a	6.1	37.50 ± 0.00 a	0.0
	1000	26.00 ± 2.34 a	−36.8	29.00 ± 1.16 a	12.1	37.50 ± 0.00 a	0.0
	1250	25.00 ± 2.50 a	−31.6	28.00 ± 1.12 a	15.2	37.50 ± 0.00 a	0.0
	1500	25.00 ± 2.00 a	−31.6	27.50 ± 1.65 a	16.7	37.50 ± 0.00 a	0.0
ML	250	32.00 ± 0.32 b	−68.4	37.50 ± 0.00 a	−13.6	37.50 ± 0.00 a	0.0
	500	30.00 ± 0.60 ab	−57.9	37.00 ± 1.48 a	−12.1	37.50 ± 0.00 a	0.0
	750	29.00 ± 1.74 ab	−52.6	36.00 ± 1.08 a	−9.1	37.50 ± 0.00 a	0.0
	1000	27.00 ± 1.08 ab	−42.1	35.00 ± 1.40 a	−6.1	37.50 ± 0.00 a	0.0
	1250	25.00 ± 2.00 ab	−31.6	34.00 ± 1.36 a	−3.0	37.50 ± 0.00 a	0.0
	1500	22.50 ± 1.58 a	−18.4	33.25 ± 0.67 a	−0.8	37.50 ± 0.00 a	0.0
EPL:COS	250	2.50 ± 0.20 d	86.8	5.25 ± 0.37 d	84.1	8.25 ± 0.66 c	78.0
	500	1.00 ± 0.05 c	94.7	2.00 ± 0.04 c	93.9	2.50 ± 0.15 b	93.3
	750	0.50 ± 0.01 b	97.4	1.00 ± 0.00 b	97.0	1.50 ± 0.15 ab	96.0
	1000	0.50 ± 0.04 b	97.4	1.00 ± 0.07 b	97.0	1.50 ± 0.02 ab	96.0
	1250	0.00 ± 0.00 a	100.0	0.50 ± 0.03 ab	98.5	1.00 ± 0.04 ab	97.3
	1500	0.00 ± 0.00 a	100.0	0.00 ± 0.00 a	100.0	0.00 ± 0.00 a	100.0
MR+COS	200	2.00 ± 0.08 c	89.5	5.00 ± 0.40 b	84.8	7.00 ± 0.56 b	81.3
	400	2.00 ± 0.06 c	89.5	5.00 ± 0.25 b	84.8	7.00 ± 0.70 b	81.3
	600	2.00 ± 0.04 c	89.5	4.00 ± 0.04 b	87.9	6.75 ± 0.20 b	82.0
	800	1.00 ± 0.01 b	94.7	2.00 ± 0.08 a	93.9	6.50 ± 0.20 b	82.7
	1000	0.00 ± 0.00 a	100.0	1.00 ± 0.02 a	97.0	5.00 ± 0.35 ab	86.7
	1200	0.00 ± 0.00 a	100.0	1.00 ± 0.05 a	97.0	4.00 ± 0.20 a	89.3
ML+COS	200	4.00 ± 0.36 d	78.9	5.00 ± 0.30 c	84.8	4.75 ± 0.38 c	87.3
	400	2.00 ± 0.20 c	89.5	3.00 ± 0.24 b	90.9	4.25 ± 0.21 c	88.7
	600	2.00 ± 0.10 c	89.5	3.00 ± 0.15 b	90.9	4.30 ± 0.09 c	88.5
	800	2.00 ± 0.08 c	89.5	3.00 ± 0.12 b	90.9	4.50 ± 0.09 c	88.0
	1000	1.00 ± 0.03 b	94.7	1.00 ± 0.08 a	97.0	2.00 ± 0.04 b	94.7
	1200	0.00 ± 0.00 a	100.0	0.00 ± 0.00 a	100.0	0.00 ± 0.00 a	100.0

COS, EPL, MR and ML stand for chitosan oligomers, ε-polylysine, *S. rochei* secondary metabolites and *S. lavendofoliae* secondary metabolites, respectively.

Table S4. Bioactive secondary metabolites produced by *S. lavendofoliae* and *S. rochei*.

Strain	Bioactive compounds	Applications	References
S. lavendofoliae	Anthracidin A	Antibiotic	[1]
	Aclacynomicin A		[2]
	Fosfazinomycins (hydrazides)	Antifungal	[3]
	Piperastatin A	Carboxypeptidase inhibitor	[4]
	Piperastatin B		[5]
	Depsidomycin	Antimicrobial and immunosuppressive activity	[6]
S. rochei	Lankacidin	Antibiotic	[7]
	Streptothricin		[8]
	Ravidomycin analogues, FE35A and FE35B	Apoptosis inducers	[9]
	Borrelidin	Antifungal	[10–12]
	Butyrolactol A		
	Butyrolactol B		
	Uricase		

References

1. Al-Humiany, A.U.-R.A.A. Taificidin1 and Taificidin2, two anti-microbial agents isolated from the fermentation broth of *Streptomyces roseodistaticus* TA15 and *Streptomyces lavendofoliae* TA17. *Res. J. of Microbiol.* **2011**, *6*, 328–342.
2. Kim, W.S.; Youn, D.J.; Kim, H.R.; Rhee, S.K.; Choi, E.S. Metabolic conversion of aclacinomycins B and Y to A by pH shift during fermentation with *Streptomyces lavendofoliae* DKRS. *Biotechnol. Tech.* **1995**, *9*, 671–676.
3. Le Goff, G.; Ouazzani, J. Natural hydrazine-containing compounds: Biosynthesis, isolation, biological activities and synthesis. *Biorg. Med. Chem.* **2014**, *22*, 6529–6544.
4. Murakami, S.; Harada, S.; Yamazaki, T.; Takahashi, Y.; Hamada, M.; Takeuchi, T.; Aoyagi, T. Piperastatin A, a new selective serine carboxypeptidase inhibitor produced by Actinomycete. I. Taxonomy, production, isolation and biological activities. *J. Enzym. Inhib.* **2008**, *10*, 93–103.
5. Murakami, S.; Harada, S.; Takahashi, Y.; Naganawa, H.; Takeuchi, T.; Aoyagi, T. Piperastatin B: A new selective serine carboxypeptidase inhibitor from *Streptomyces lavendofoliae* MJ908-WF13. *J. Enzym. Inhib.* **2008**, *11*, 51–66.
6. Narayanaswamy, V.K.; Albericio, F.; Coovadia, Y.M.; Kruger, H.G.; Maguire, G.E.M.; Pillay, M.; Govender, T. Total synthesis of a depsidomycin analogue by convergent solid-phase peptide synthesis and macrolactonization strategy for antitubercular activity. *J. Pept. Sci.* **2011**, *17*, 683–689.
7. Arakawa, K.; Sugino, F.; Kodama, K.; Ishii, T.; Kinashi, H. Cyclization mechanism for the synthesis of macrocyclic antibiotic Lankacidin in *Streptomyces rochei*. *Chem. Biol.* **2005**, *12*, 249–256.
8. Anukool, U.; Gaze, W.H.; Wellington, E.M.H. In situ monitoring of Streptothricin production by *Streptomyces rochei* F20 in soil and rhizosphere. *Appl. Environ. Microbiol.* **2004**, *70*, 5222–5228.
9. Yamashita, N.; Shin-Ya, K.; Furihata, K.; Hayakawa, Y.; Seto, H. New Ravidomycin analogues, FE35A and FE35B, apoptosis inducers produced by *Streptomyces rochei*. *J. Antibiot.* **1998**, *51*, 1105–1108.
10. Kanini, G.S.; Katsifas, E.A.; Savvides, A.L.; Karagouni, A.D. *Streptomyces rochei* ACTA1551, an indigenous Greek isolate studied as a potential biocontrol agent against *Fusarium oxysporum* f. sp. lycopersici. *BioMed Res. Int.* **2013**, *2013*, 1–10.
11. Augustine, S.; Bhavsar, S.; Kapadnis, B. Production of a growth dependent metabolite active against dermatophytes by *Streptomyces rochei* AK 39. *Indian. J. Med. Res.* **2005**, *121*, 164–170.
12. Irdani, T.; Perito, B.; Mastromei, G. Characterization of a *Streptomyces rochei* endoglucanasea. *Ann. N.Y. Acad. Sci.* **1996**, *782*, 173–181.

© 2019 by the authors. Licensee MDPI, Basel, Switzerland. This article is an open access article distributed under the terms and conditions of the Creative Commons Attribution (CC BY) license (http://creativecommons.org/licenses/by/4.0/).
